# Lipid and lipoprotein profiles among middle aged male smokers: a study from southern India

**DOI:** 10.1186/1617-9625-8-11

**Published:** 2010-10-05

**Authors:** Ramachandran Meenakshisundaram, Chinnasamy Rajendiran, Ponniah Thirumalaikolundusubramanian

**Affiliations:** 1 Madras Medical College, Chennai, India; 2 Chennai Medical College Hospital & Research Center, Irungalur, Trichy, India

## Abstract

**Objectives:**

The objectives were to investigate into the relationship between lipid profile including Apolipoprotein-A1 (Apo-A1) and Apolipoprotein-B (Apo-B) and smokers and to relate them with smoking pack years.

**Materials and Methods:**

A total of 274 active male smokers without any other illnesses and age matched male healthy control subjects (78) with similar socio-cultural background were assessed for clinical details, dietary habits, physical activities, smoking and alcohol consumption. Standard methods were adopted to check the lipid levels. The data were analyzed statistically.

**Results:**

Their ages ranged from 40 to 59 years, systolic BP from 110 to 130 mmHg, and diastolic BP from 76 to 88 mmHg. All of them had similar pattern of diet (vegetarianism with occasional meat). None was on any medication influences lipid level. Their physical activity was moderate. Number of pack years varied from 10 to 14 (mild), 15 to 19 (moderate) and 20 and above (heavy) among 69, 90 and 115 cases, whose mean ages were 43, 44 and 49 respectively. The mean (+SD) values in mg/dl of total cholesterol (TC), Triglyceride (TGL), Apo-B, low density lipoprotein (LDL) cholesterol, high density lipoprotein (HDL) cholesterol and Apo-A1 in mg/dl among mild/ moderate/ heavy smokers and control subjects were 198 (30.6)/ 224 (27.2)/ 240 (24.3) and 160 (20.4); 164(42.6)/ 199 (39.5)/ 223(41.7) and 124 (31.6); 119 (24.9)/ 121 (27)/ 127 (28.3) and 116 (21.4); 94 (19.7)/ 104 (21.8)/ 120 (20.5) and 82 (17.6); 42 (5.9)/ 39 (3.1)/ 35(4.4) and 48 (5.3); and 120 (17)/ 119 (21)/ 115 (25) and 126 (19), respectively. In smokers, there was a rise in TC, TGL, LDL, Apo-B and fall in HDL and Apo-A; these changes were significant (P < 0.05).

**Conclusion:**

Number of pack years was directly proportional to abnormal lipid profile. It is also concluded that changes in Apo-A1 and Apo-B were more significant when compared to HDL and LDL cholesterol among smokers. In the view of double risk for smokers (smoking and altered lipid profile) efforts may be made to introduce smoking cessation program.

## Introduction

Smoking is an escalating health problem especially in developing countries such as India. Cigarette smoking is a known risk factor for peripheral, coronary and cerebral atherosclerotic vascular diseases. Cigarette smoking leads to the uptake of many hazardous compounds and their metabolites extracted from burning tobacco. These substances may be electrophilic and react with biological molecules, and give rise to oxidative stress through the formation of reactive species or the initiation of lipid peroxidation chain reactions in the membranes[1]. Plasma lipoprotein abnormalities are major risk factor for the occurrence of atherosclerotic vascular disease [2]. The prevalence of smoking in India varies from about 15% to over 50% among men [3]. However, smoking is less common among women with prevalence of 4% or less [4]. Cigarette smoking has been found to alter the lipoprotein levels [5].

Previously published reports suggest their oxidatively modified low density lipoprotein (LDL) is taken up by macrophages to form foam cells in culture and aggravate the process of atherosclerosis[6]. Also, the effects of elevated lipid levels and changes in lipoprotein among cigarette smokers were demonstrated earlier [7], [8-10]. The effects of cigarette smoking on serum apolipoprotein A1 (Apo A1) and apolipoprotein B (Apo B) in smokers free from other risk factors of atherosclerotic vascular disease and dose response relationship were studied. The correlation of Apo A1 with high density lipoprotein cholesterol (HDL) and Apo B with LDL as coronary risk factors was also examined along with the effects of smoking on HDL/Apo A1 and LDL/Apo B. However, studies on the interaction between smoking and apolipoproteins are scarce. Hence, objectives of the present study were: (i) to investigate into the lipid profile including Apo-A1 and Apo-B among smokers, and (ii) to relate lipid profile alteration with smoking pack years.

## Materials and methods

### Selection of subjects

Middle aged healthy males smoking cigarettes over 10 pack years and hailing from Chennai, capital of Tamil Nadu State of Southern India attending Master health checkup program (MHCP) formed the subjects of the study. Another group of non-smoking age matched male candidates attending MHCP were included as control subjects. The work was carried out after an approval from Institutional ethical committee and informed consent from each participant according to Helsinki Declaration Guidelines.

### Master health checkup program (MHCP)

State Government of Tamil Nadu has introduced MHCP in Government Medical College hospital and district head quarters hospitals wherein any interested or referred persons can ask for health check-up. Subjects are instructed to come in fasting for 12 hours. During checkup, qualified physician takes complete health history including health complaints, past, family and social histories, and physician does general and physical examination of body systems. Then, individuals are subjected to laboratory evaluation such as complete blood count, blood sugar, lipid profile, renal and liver function, and urine analysis including chest X-ray, ultra sonogram of abdomen and electrocardiogram for a nominal fee. If the individual is found to have any illness, he/she will be referred to respective specialty for management. Counseling and guidance will be provided to those who require life style modification.

### Inclusion and exclusion criteria

Since smoking is extremely rare among women in this area due to cultural reasons, women were not included. Individuals if found to have any associated co-morbid illness or taking regular medication including vitamin/mineral supplements/herbal/native medicines were excluded. Clinical history was elicited to rule out any acute injury or infectious episode and/or anti-microbial therapy over the last six weeks. Patients with family history of lipid disorders were not included.

### Data collection

The socio-demographic and clinical data were collected. Dietary history and physical activity were elicited as per Indian Council of Medical Research.

### Categorization of smokers

Smoking history was elicited in detail and smoking pack year was then calculated by using formula, {(Number of cigarettes smoked per day × Number of years smoked)/20}. In our study, smokers were classified into mild, moderate and heavy based on the number of pack years as 10 to 14, 15 to 19, and 20 and above, respectively.

### Laboratory aspects

Blood was drawn from the subjects after 12 hours fasting with staple food for two days. Enzymatic method was used to estimate total cholesterol (TC) and triglycerides (TGL) using commercial kits. HDL cholesterol was determined by precipitation of phosphotungstic acid MgCl [11] and LDL cholesterol was then calculated. By kinetic nephelometry, Apo-A1 and Apo-B were measured using specific antibodies.

### Statistical analysis

The data were tabulated and analyzed. Differences between mean values were evaluated by student 't' test. The statistical significance was assessed by using chi-square test [12].

## Results

There were 274 smokers and 78 controls. Their ages ranged from 40 to 59 years. Their systolic blood pressure and diastolic blood pressure ranged from 110 to 130 and 76 to 88 mmHg, respectively. Most of them were vegetarians with occasional meat (varied from 3 to 5 times per month) and rice was their staple food. The subject groups consumed coffee or tea from 2 to 3 times per day. All subject groups had moderate physical activity of at least 30 minutes per day. Overall, there was no difference noticed among smokers and non-smokers with reference to diet, physical activity and life style except smoking. Among smokers the number of pack years varied from 10 to 14, 15 to 19, and 20 and above among 69, 90 and 115 cases, whose median ages were 43, 44 and 49 respectively. The mean values of TC, TGL, LDL, HDL, Apo-B and Apo-A1 for smokers and non-smokers (control subjects) are depicted in Table 1. Among the smokers TC, LDL, TGL and Apo-B were elevated significantly than control (P < 0.05). HDL and Apo-A1 were not statistically reduced in mild smokers. Among moderate smokers, HDL was not significantly reduced (odds ratio (OR) = 1.81, 95% confidence interval (CI) = 0.82-4.02, P > 0.05), however Apo-A1 was significantly reduced (OR = 2.72, 95% CI = 1.45-5.10, P < 0.05). In heavy smokers Apo-A1 association (OR = 3.33, 95% CI = 1.82-6.10, P < 0.05) was stronger than HDL (OR = 4.30, 95% CI = 1.73-10.67, P < 0.05). Similarly LDL and Apo-B were significantly increased among all groups of smokers (P < 0.05 to 0.00). Concordant increase of Apo-B with LDL and concordant decrease of Apo-A1 and HDL were noticed among smokers (P < 0.05). The abnormalities of lipid profile were more significant when smoking pack year increased. The statistical significance of lipid profile among smokers is illustrated in Table 2.

**Table 1:** Lipid Profile among Smokers and Control subjects:

Lipid (in mg/dl)	Control, N = 78, Mean ± SD	Mild (Pack years 10-14) N = 69, Mean ± SD	Moderate (Pack years 15-19) N = 90, Mean ± SD	Heavy (Pack Years≥20) N = 115, Mean ± SD
TC	160 ± 20.4	198 ± 30.6#	224 ± 27.2#	240 ± 24.3#
TGL	124 ± 31.6	164 ± 42.6*	199 ± 39.5#	223 ± 41.7#
LDL-C	82 ± 17.6	94 ± 19.7#	104 ± 21.8#	120 ± 20.5#
HDL-C	48 ± 5.3	42 ± 5.9	39 ± 3.1	35 ± 4.4*
Apo-B	116 ± 21.4	119 ± 24.9#	121 ± 27#	127 ± 28.3#
Apo-A1	126 ± 19	120 ± 17	119 ± 21*	115 ± 25#

# P < 0.001; *P < 0.05; TC = total cholesterol, TGL = triglyceride, LDL-C = low density lipoprotein cholesterol, HDL-C = high density lipoprotein cholesterol, Apo-B = Apolipoprotein B, Apo-A1 = Apolipoprotein A1, SD = standard deviation, N = number.

**Table 2:** Odds Ratio (95% Confidence Interval) for Lipid Profile among Smokers:

Lipid (in mg/dl)	Mild (Pack years 10-14)	Moderate (Pack years 15-19)	Heavy (Pack years ≥ 20)
TC (≥ 200)	6.26 (3.04-12.90)#	10.25 (4.94-21.29)#	14.82 (7.10-30.92)#
TGL (≥ 150)	3.13 (1.46-6.70)*	3.91 (1.87-8.13)#	5.67 (2.70-11.87)#
LDL-C (≥ 130)	3.99 (1.20-7.97)#	6.07 (3.06-12.01)#	13.01 (6.19-27.33)#
HDL-C (≤ 40)	1.64 (0.71-3.82)	1.81 (0.82-4.02)	4.30 (1.73-10.67)*
Apo-B (> 110)	6.64 (3.17-13.90)#	9.17 (4.44-18.95)#	17.74 (8.08-38.85)#
Apo-A1(< 110)	1.19 (0.63-2.28)	2.72 (1.45-5.10)*	3.33 (1.82-6.10)#

# P < 0.001; *P < 0.05; TC = total cholesterol, TGL = triglyceride, LDL-C = low density lipoprotein cholesterol, HDL-C = high density lipoprotein cholesterol, Apo-B = Apolipoprotein B, Apo-A1 = Apolipoprotein A1.

## Discussion

Previous studies have demonstrated a rise in TC, TGL, LDL and Apo-B, and a fall in HDL and Apo-A in smokers; and this association is dose dependant [9,10,13][14,15]. It has been suggested that smoking, even of short duration and moderate consumption of cigarettes, is associated with adverse lipoprotein profiles [16]. Apolipoproteins are known to determine the structural stabilities and metabolic directions of lipoproteins. Of the apolipoproteins, Apo-B has identified in VLDL and LDL and thus appears to be a measure of the total number of atherogenic particles [17]. Hence, Apo-B is a more accurate measure than LDL with regard to predicting the CAD. Though LDL and Apo-B were significantly elevated among all three categories of smoker, Apo-B elevation was much stronger than LDL. In moderate smokers, only Apo-A1 was significantly decreased without significant reduction in HDL level. Apo-A1 association was stronger than HDL in heavy smokers. Hence, we conclude that changes in Apo-A1 and Apo-B were significant when compared to HDL and LDL cholesterol among smokers. Thus, it is clear that alteration in apolipoproteins occurs earlier than its corresponding cholesterol, which concurs with previous observation [18]. Such similar observation in lipid profile, such as rise in TC, TGL and LDL and fall in HDL were noted among passive smokers [8,19].

Smoking is associated with coronary artery disease (CAD) and other vascular disorders, cancer, chronic obstructive pulmonary disease (COPD) etc., Several mechanisms were proposed to explain the deleterious effect of tobacco. For the occurrence of cardiovascular disease among smokers alteration in plasma lipid profile was implicated. In this context, the mechanisms for the altered lipid profile among smokers were recalled [5].

(i) Nicotine stimulates the release of adrenaline from the adrenal cortex leading to increased serum concentration of free fatty acids (FFA)which further stimulates hepatic synthesis and secretion of cholesterol [20] as well as hepatic secretion of very low density lipoprotein (VLDL) and hence increased TGL [21].

(ii) Smoking decreases estrogen levels and further leads to decreased HDL cholesterol concentration [22,23]. Also, HDL concentration was inversely related to VLDL concentration in serum.

(iii) Smoking increases insulin resistance and thus, causes hyperinsulinemia. LDL, VLDL and TGL are elevated in hyperinsulinemic conditions due to decreased activity of lipoprotein lipase [23].

Inconsistent observations were noticed for smoking mediated LDL change. Some suggested that the effect of smoking on LDL is mediated through reduction in lipoprotein lipase [24,25] which was contradicted by Moriguchi and Eliasson et al., that no difference in lipoprotein lipase activity observed between smokers and non-smokers [26,27]. Plasma lipase is an important regulator of plasma lipoprotein concentration. TGL rich lipoprotein is hydrolyzed by the catalyst lipoprotein lipase and thus, enables clearance of TGL from blood. Among smokers, hepatic lipase has been activated [26], which converts VLDL to LDL[28]. In an experimental study, it has been shown that smoking leads to inhibition of lecithin-cholesterol acyl transferase [29]. Nicotine also exerts hyperlipidemic effects by increasing the synthesis of TGL rich lipoprotein[30]. It is also interesting to note that smoking induces oxidative stress resulting in glutathione peroxidase [GSH-Px] activity where it increases glutathione reductase (GR) activity in erythrocytes. Also, human serum paraoxygenase (PON1) is a polymorph enzyme which has been shown to play an important role in lipid metabolism. PON1 significantly decreases lipid peroxidase generation during LDL oxidation in the presence HDL modification by lipid peroxidase [31,32]. Smoking impairs PON1 activity and thereby compromises anti-oxidant defense mechanism [33].

Smokers had significantly higher level of Apo-B [34,35] and higher level of Apo-B is believed to be related to the risk of premature CAD [36]. Though the smokers are considered for the present study did not show any symptomatic evidence of CAD, increased levels of Apo-B in them may be a warning sign long before the onset of symptoms of CAD. In smokers, low level of Apo-A1 has been associated with significant atherosclerotic changes in patients undergoing coronary angiography [37]. Moreover, the ratio of Apo-B to Apo-A was significantly higher in smokers regardless of sex [38]. As further evidence in support of casual relation lipid and lipoprotein concentration in ex-smokers were either the same as those found in non-smokers or were intermediate between smokers and non-smokers [5]. In a longitudinal study, it was observed that HDL and Apo-A1 levels in ex-smokers return towards base line concentration seen in non-smokers [39]. Because of reversible phenomenon, occurrence of lipid profile alteration and its atherosclerotic complications come down among middle aged smokers after cessation of cigarette smoking.

## Conclusion

Abnormalities in lipid profile are directly correlated with smoking and duration of smoking pack years in this study. Since apolipoprotein changes occur earlier than cholesterol level, it would be advisable to include apolipoprotein concentration in lipid panel. Further studies are warranted to establish the role of apolipoprotein concentration in patients on lipid lowering therapy as a guide for response to therapy and genomic factors in apolipoprotein synthesis. Cessation of smoking not only reverses lipid changes but also vascular diseases, especially CAD. Passive smokers are prone to get the same abnormality as demonstrated in literature and our on-going analysis. Hence, policy makers should amend a firm law to prohibit smoking in the public places, thereby decreasing the passive smokers in the community. Intense education program about adverse health events of smoking should be under taken through all means including audio-visual media to the public and to students through their curriculum.

## Strengths and limitation

Strength of the study was rigid criteria adopted to select the subjects. Limitation being the study was confined to males of specific age group belonging to the same geographical area.

## Competing interests

The authors declare that they have no competing interests

## Authors' contributions

RM carried out study design, study protocol, sample collection, statistical analysis, references collection and manuscript drafting. CR and PT took part in study design, study protocol, approval, revision of statistical work and manuscript. All authors read and approved the final manuscript.
